# Cucurbitacin B induces neurogenesis in PC12 cells and protects memory in APP/PS1 mice

**DOI:** 10.1111/jcmm.14514

**Published:** 2019-06-30

**Authors:** Jing Li, Kaiyue Sun, Makoto Muroi, Lijuan Gao, Young‐Tae Chang, Hiroyuki Osada, Lan Xiang, Jianhua Qi

**Affiliations:** ^1^ College of Pharmaceutical Sciences Zhejiang University Hangzhou China; ^2^ Chemical Biology Research Group RIKEN Center for Sustainable Resource Science Saitama Japan; ^3^ Center for Self‐assembly and Complexity Institute for Basic Science (IBS) Pohang Korea; ^4^ Department of Chemistry Pohang University of Science and Technology (POSTECH) Pohang Korea

**Keywords:** Alzheimer's disease, cofilin, cucurbitacin B, glucocorticoid receptor, TrkA

## Abstract

Cucurbitacin B (CuB) isolated from *Cucumis melo* by using a PC12 cell bioassay system exhibited significant nerve growth factor (NGF)‐mimic or NGF‐enhancing activity in PC12 and primary neuron cells. It was also demonstrated pro‐neurogenesis effects in ICR and APP/PS1 mice and improved memory deficit of APP/PS1 mice. Its possible mechanism includes significant induction of the phosphorylation of glucocorticoid receptor (GR), protein kinase C (PKC), phospholipase C (PLC) and inhibition of cofilin. ChemProteoBase profiling, binding assay and cellular thermal shift assay (CETSA) were used to determine the target protein. Results revealed that CuB could affect actin dynamics as an actin inhibitor but did not bind with GR. The protein level of cofilin in PC12 cells after treating 0.3 μM and different temperatures was significantly higher than that of control group. Other neurotrophic signalling pathways, such as TrkA/TrkB, were analysed with specific inhibitors and Western blot. The inhibitors of TrkA, PLC, PKC, Ras, Raf and ERK1/2 significantly decreased the percentage of PC12 cells with neurite outgrowth and shortened the length of neurite outgrowth induced by CuB. CuB significantly induced the phosphorylation of TrkA, ERK and CREB. The phosphorylation of these proteins was obviously decreased by their specific inhibitors. These results suggest that cofilin is a candidate target protein of CuB in PC12 cells and that the GR/PLC/PKC and TrkA/Ras/Raf/ERK signalling pathways play important roles in the neuroprotective effect of CuB.

## 1 INTRODUCTION

Alzheimer's disease (AD) is a progressive neurodegenerative disease. The World Alzheimer Report 2016 described that about 47 million people were suffering from dementia. The pathophysiology of AD includes neurotransmitter deficiency, Aβ protein accumulation, neurofibrillary tangles, neuron loss and so on.^1^ Among these mechanisms, neuron loss is one of the major disease risks of AD.^2^ Neuroscience research shows that the generation of newborn neurons is sustained throughout adulthood because of the proliferation and differentiation of adult neural stem cells. Neural stem cells in the subgranular zone of the hippocampus facilitate the formation of new neurons that migrate a short distance into the granule cell layer of the dentate gyrus (DG), which shows lifelong structural and functional plasticity.^3^ However, the survival of newborn neuronal cells in AD transgenic mice that exhibit Alzheimerr's‐type amyloid pathology is dramatically impaired. Thus, attempts to increase the number of newborn neuron cells in the hippocampus have been exerted to improve memory, which is crucial for the prevention or treatment of AD.^4^

The PC12 cell bioassay system is proven reliable in the investigation of potential drugs with nerve growth factor (NGF)‐mimic activity.^5^ NGF is a secreted growth factor that is important in the survival, growth and maintenance of specific types of neurons in the central nervous system (CNS) and peripheral nervous system.^6^ Similar to neuron cells, PC12 cells cease dividing and begin to differentiate after treatment with NGF. However, the application of NGF as a drug is limited by its inability to cross the blood‐brain barrier (BBB). Thus, finding novel molecules that demonstrate NGF‐mimic activity, exhibit neurogenesis function and can cross the BBB is important.^5^

Traditional Chinese medicines (TCMs) are great sources of compounds with medical uses. TCMs include many potential lead compounds that display NGF‐mimic or NGF‐enhancing activity in PC12 cells.^5^, ^7^ Cucurbitacin B (CuB) isolated from *Cucumis melo* (Tiangua Di in Chinese) shows NGF‐mimic activity in PC12 cells. CuB also exhibits pharmacological functions, such as anti‐inflammatory, analgesic and antiplasmodial activities.^8^, ^9^ Furthermore, CuB has been approved by the State Food and Drug Administration (SFDA) and prescribed to treat hepatitis and hepatic carcinoma. However, reports about the in vivo and in vitro neurogenesis and neuroprotective activity of CuB are lacking.

The present study revealed the neuroprotective function of CuB. This function is mainly mediated by glucocorticoid receptor (GR) signalling pathway, and cofilin is considered as the candidate target. At the same time, we find that TrkA/Ras/Raf/extracellular signal‐regulated kinase (ERK) is also involved in the function of CuB.

## 2 MATERIALS AND METHODS

### 2.1 Isolation and structure elucidation of CuB

**Figure 1:**
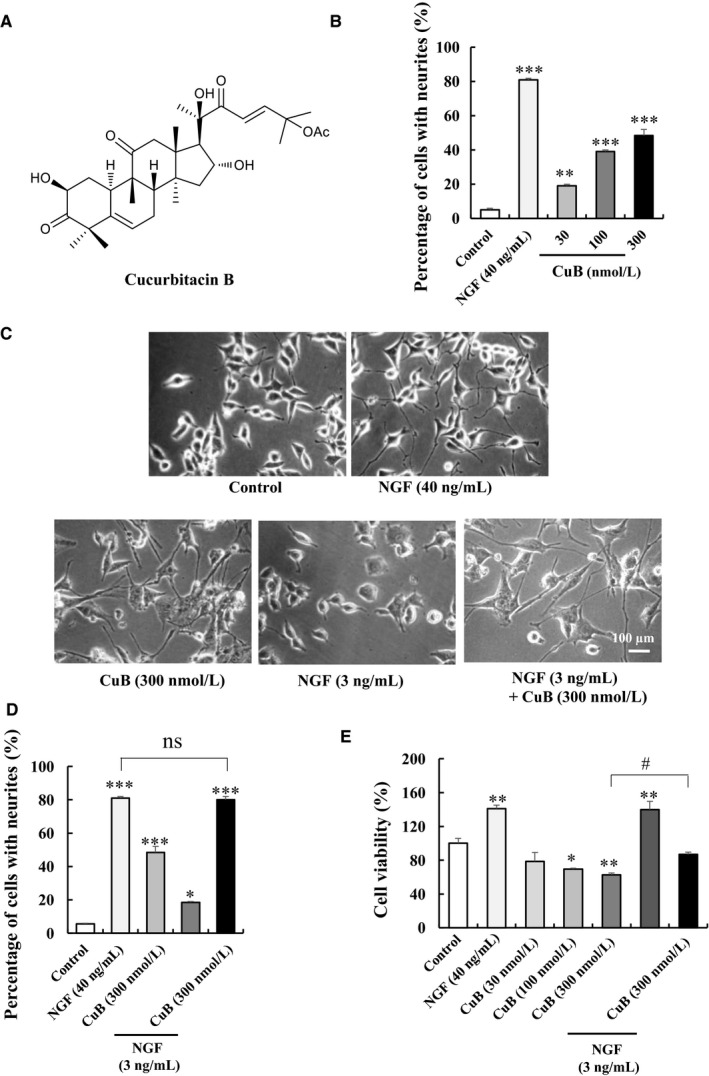
Neurogenesis effect of cucurbitacin B (CuB) on PC12 cells. A, Chemical structure of CuB. B, Percentage of neurite outgrowth of PC12 cells after treatment with 30, 100 and 300 nM CuB for 48 h. C‐D, Morphological change and percentage of neurite outgrowth of PC12 cells treated with CuB and NGF. Scale bar is 100 µm. E, MTT assay result of different doses of CuB and CuB with 3 ng/mL NGF in PC12 cells. Each experiment was repeated for three times. The data represented mean ± SEM. *P < 0.05, **P < 0.01 and ***P < 0.001 indicated significant difference compared with the control group; ^#^ *P* < 0.05 indicated significant difference compared with 300 nM CuB group; ns indicated no significant difference compared with 40 ng/mL NGF group

Cucurbitacin B (Figure 1A) was isolated from the stems of *C melo*, and the chemical structure was determined by comparing ^1^H NMR and ^13^C NMR with the reported literature.^10^ Detailed separation and structure elucidation steps are presented in supplementary materials.

### 2.2 NGF‐mimic activity bioassay in PC12 cells

Normal‐type PC12 cell line was purchased from the Cell Bank of the Chinese Academy of Sciences (Shanghai, China). Meanwhile, wide‐type and Ras mutant‐type PC12 cells (RasN17) were provided by Professor Hiroyuki Osada.^11^ In NGF‐mimic assay, 20 000 PC12 cells were seeded in each well of a 24‐well microplate in 1 mL CM medium (Dulbeccor's modified Eagler's medium [DMEM; Thermo Scientific] containing 10% foetal bovine serum, 5% horse serum and 1% pre‐mixed antibiotics [Invitrogen]) and incubated in 5% CO_2_ at 37°C. The medium was replaced after 24 hours with 1 mL serum‐free DMEM medium containing a test sample or 0.5% dimethyl sulphoxide (DMSO). In the inhibitor test, the cells in 24‐well microplates were pre‐cultured with 500 μL medium containing an inhibitor for 30 minutes. Then, another 500 μL medium containing a test sample or 0.5% DMSO was added. The inhibitors of TrkA, ERK, GR, PLC, PKC and Ras (K252a, U0126, RU486, U73343, GO6983 and S3131) were obtained from Sigma. The Raf and TrkB inhibitors (AZ628 and ANA‐12) were purchased from Axon Medchem BV and Selleckchem, respectively. After 48 hours, the morphological changes in the cells were observed using a phase‐contrast microscope (Model CKX41; Olympus). About 100 cells were counted in each of three randomly chosen fields. A positive cell was defined as the neurite outgrowth of a cell longer than the diameter of cell body.

### 2.3 MTT assay

Cell viability was determined based on mitochondria‐dependent reduction of 3‐(4,5‐dimethylthiazol‐2‐yl)‐2,5‐diphenyl tetrazolium bromide (MTT), and the method of MTT assay was performed as described in other reports, and details are presented in supplementary materials.^12^

### 2.4 Primary cortical neuron cell culture and bioassay

The primary cortical neuron cells were obtained from the embryos of ICR mice, and NeuO was used to visualize the neuron cells as described in the report.^13^ Detailed methods for primary neuron cell culture and bioassay are presented in supplementary materials.

### 2.5 ChemProteoBase profiling

ChemProteoBase profiling was performed as described in other reports.^14^ Briefly, HeLa cells were first treated with CuB (1 µM) for 18 hours. A 2‐D DIGE system (GE Healthcare) was used to perform the proteome analysis of cell lysates, and images of the gels were analysed using Progenesis SameSpots (Nonlinear Dynamics). Out of more than 1000 spots detectable in each gel, 296 spots that were found in common between gels of reference compound‐treated cells were selected. The volume of each spot was then normalized using the average of the corresponding control values from DMSO‐treated HeLa cells. Using the normalized volume of the 296 spots, we calculated the cosine similarity between compounds and performed hierarchical clustering analysis with Gene Cluster 3.0 (clustering method; centroid linkage with means of uncentred correlation). The predictive dendrogram was visualized using Java Treeview 1.1.3.

### 2.6 Western blot

Briefly, 2 × 10^6^ PC12 cells were seeded and cultured in a 6‐cm plate containing 5 mL of DMEM medium for 24 hours. In the time‐dependent study of CuB, 300 nM CuB was added and incubated for designed time. In the inhibitor test, the PC12 cells were first incubated with 4 mL of DMEM medium containing an inhibitor for 30 minutes and then 1 mL of medium with test samples was added. To prepare protein lysates, the cells were collected and lysed in lysis buffer. After centrifugation at 13 800 *g* for 15 minutes, the supernatant was removed. Protein samples from PC12 cells were treated and analysed through electrophoresis as described before.^5^ Method details for Western blot are presented in supplementary materials.

### 2.7 LanthaScreen TR‐FRET competitive binding assay

The LanthaScreen TR‐FRET GR competitive binding assay was used to determine whether CuB was potential GR ligand, and method details are presented in supplementary materials.

### 2.8 Cellular thermal shift assay

The cellular thermal shift assay was performed as described in other reports.^15^ At first, 2 × 10^6^ cells were separately added into 6‐cm dishes contained 5 mL DMEM and incubated for 24 hours. After that, CuB was added into each plate at the final concentration of 0.3 µM. The cells were collected after continual incubating 0.5 hour and heated at different temperatures such as 50, 54, 58 and 62°C. Next, Western blot analysis was used to detect the change in cofilin protein as described in Western blot.

### 2.9 Animal experiments

One hundred male ICR mice (12 weeks old, 40‐45 g) and ten female ICR mice (8 weeks old, 25‐30 g) were purchased from SLAC Laboratory Animal Company. In addition, fifty APP/PS1 mice (4 months) and thirty C57BL/6J mice (4 months) were purchased from the Model Animal Research Center of Nanjing University. Mice were housed as five mice per cage, allowed free access to water and food, and maintained in constant temperature (23 ± 1°C) and humidity (55% ± 5%) under a 12‐hour light/dark cycle (lights on 8:00 to 20:00). All experiments were conducted in accordance with the Committee on Animal Experiments at Zhejiang University (Permit ZJU20160236).

### 2.10 BrdU or BrdU and NeuN immunostaining in ICR mice

To investigate the neurogenesis function of CuB in normal mice, 24 ICR mice were equally divided into control and CuB 0.02, 0.1 and 0.5 mg/kg groups. CuB was dissolved in saline with 1% Tween‐80 and 2% ethanol. The schedule of animal experiment is presented in Figure S1A. In BrdU immunostaining test, ICR mice were oral‐administered with vehicle or CuB for 31 consecutive days. Two days later, mice were received four injections of 100 mg/kg BrdU (Sigma) with an interval of 12 hours. At the end of experiment, the mice were anaesthetized with chloral hydrate (400 mg/kg, i.p.) and perfused transcardially with 4% paraformaldehyde. Then, the brains were post‐fixed for 48 hours in 4% paraformaldehyde, dehydrated for 3 days in 30% sucrose solvent followed by embedded in OCT compound (Sakura Finetek) at −30°C. Coronally, slide was cut into 50‐μM‐thick sections using a vibrating microtome (Leica CM1900). Sections were treated with formamide mixed with 4× SSC buffer for 2 hours at 65°C, and then treated with 2 M HCl for 30 minutes at 37°C, and 0.1 M boric acid (pH = 8.5) for 10 minutes at room temperature, successively. After washing, the sections were treated with goat serum for 1 hour, and then incubated with anti‐BrdU antibody (Abcam) at 4°C for 48 hours. Finally, the sections were incubated with CY3‐labelled anti‐rat IgG antibody (Millipore) in the dark for 4 hours. Afterwards, they were mounted onto subbed slides and cover‐slipped using fluoromount (Sigma). BrdU cells in bilateral hippocampal DG were assessed under a confocal laser‐scanning microscope (LSM510; Zeiss) at excitation of 488 nm.

Based on the result in Figure S1B, the most suitable concentration of CuB is 0.1 mg/kg. Thus, 0.1 mg/kg CuB was used to investigate the newborn neurons in ICR mice by BrdU/NeuN double immunostaining as shown in Figure S1A. The protocol is similar with BrdU immunostaining test, except that the sections were incubated with both anti‐BrdU and anti‐NeuN (Millipore) antibodies followed by incubation with both CY3‐labelled anti‐rat IgG antibody and fluorescent‐conjugated antimouse antibody (Millipore).

### 2.11 Neuroprotection experiment of CuB on the APP/PS1 mice

The neuroprotection experiment of CuB in APP/PS1 mice was performed as presented in Figure S1C. Mice were divided into three groups: (a) C57BL/6J control; (b) APP/PS1 control; and (c) APP/PS1 + CuB (0.1 mg/kg), each group had eight mice. The experiment included animal behaviour tests and BrdU/NeuN double immunostaining test. Two control groups received vehicle, whereas the APP/PS1 + CuB (0.1 mg/kg) group received 0.1 mg/kg CuB for consecutive 31 days. After 2 days of oral administration, mice were received four injections of 100 mg/kg BrdU with an interval of 12 hours. Then, 19 days later, mice were subjected to the Y‐maze, novel objects recognition (NOR) and Morris water maze (MWM). Finally, the brains of mice were obtained and used for the BrdU/NeuN double immunostaining test, and the method was the same as that in the double immunostaining study on ICR mice.

### 2.12 Animal behaviour tests

The Y‐maze is a three‐arm maze with equal angles between all arms, which were 30 cm long and 5 cm wide with 12‐cm‐high walls. The mice were initially placed in one arm and allowed to move freely. The sequence and number of arm entries were manually recorded over 8‐minute periods. Alternation was considered a successive entry when the mice entered all three arms successively, that is BCA but not ABA. The spontaneous alternation (%) was defined as the total number of alternations divided by the total number of entries.

The apparatus of NOR task contains an open‐field arena of 40 cm × 25 cm × 20 cm (length × width × height), one novel object and two familiar objects. In the first 2 days, mice were placed into the apparatus without object for 5 minutes for habituation. The training and test sessions started in the third day. In the training session, mice were placed in the apparatus containing two familiar objects and allowing exploring for 5 minutes. The objects were positioned in two adjacent corners, 5 cm from the walls. The test session was performed 1 hour after training session, in which one familiar object was replaced with a novel object. Mice were allowed to explore the objects for 5 minutes. Exploration time was defined by directing the nose to the object at a distance within 2 cm and/or touching the object with the nose or forepaws. The object recognition index (%) was represented with the percentage of exploring time on the novel object divided by the total exploring time.

The MWM was performed as other reports,^16^, ^17^ and the detail description is displayed in the supplementary information.

### 2.13 Statistical analysis

All experiments were independently performed two or three times, and each experiment was conducted using six or eight samples. Data were presented as mean ± SEM. Significant differences between groups were determined by ANOVA, followed by one‐tailed Tukey's multiple comparison test using GraphPad Prism 5.0 software (GraphPad Software). Statistical significance was considered at *P* < 0.05.

## 3 RESULTS

### 3.1 CuB increases neurite outgrowth and length in PC12 cells and primary neuron cells in vitro

The neurogenesis effect of CuB was first confirmed in PC12 cells in vitro. Cells were treated with CuB at various concentrations of 30, 100 and 300 nM for 48 hours. CuB from 30 to 300 nM could dose‐dependently increase neurite outgrowth and length in PC12 cells (Figure 1B and Figure S2A, *P* < 0.01, *P* < 0.001, *P* < 0.001). Interestingly, CuB‐combined NGF could markedly increase the percentage of PC12 cells with neurite outgrowth from 48.5 ± 3.5% to 80.0 ± 2.0% (Figure 1C‐D) and neurite length of PC12 cells from 335.3 ± 28.8 to 420.9 ± 28.5 µm (Figure S2B). These data indicate that CuB exhibits a novel NGF‐mimic and NGF‐enhancer ability in PC12 cells. To evaluate the cytotoxicity of CuB at the cellular level, MTT assay was conducted. The viabilities of PC12 cells were 78.7 ± 10.3%, 69.4 ± 1.4% and 62.6 ± 2.5% after treating CuB at doses of 30, 100 and 300 nM (*P* < 0.05, *P* < 0.01), respectively. These results indicate that CuB has cytotoxic to PC12 cells. However, the viability of PC12 cells in 300 nM CuB‐treated group was significantly increased to 86.9 ± 2.5% after adding NGF at a dose of 3 ng/mL (*P* < 0.05). This result suggests that low‐dose NGF can lower the toxicity of CuB.

**Figure 2:**
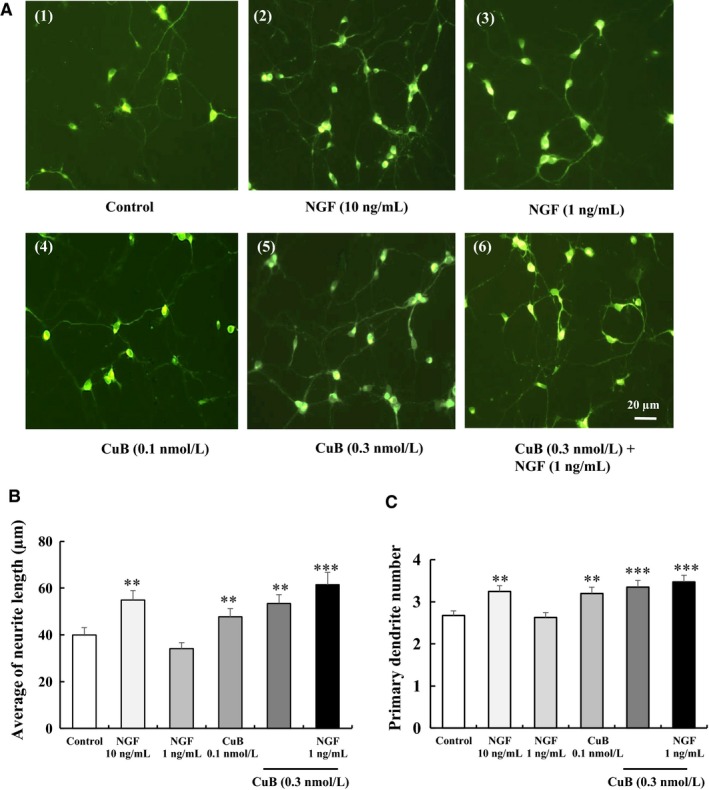
Neurogenesis effect of cucurbitacin B (CuB) on primary neuron cells. A, Morphological changes in primary neuron cells after treatment with agents for 72 h: (1) control (0.5% DMSO), (2) positive control (NGF 10 ng/mL), (3) NGF 1 ng/mL, (4) CuB 0.1 nM, (5) CuB 0.3 nM and (6) CuB 0.3 nM + NGF 1 ng/mL. Scale bar is 20 µm. B‐C, The average of neurite length and primary dendrite number in each group. For each result, 60 cells were counted. This experiment was repeated for three times. The data represented mean ± SEM. **P < 0.01, ***P < 0.001 indicated significant difference compared with the control group

The neurogenesis effect of CuB was then estimated in primary neuron cells. As shown in Figure 2A, the neurite outgrowth of primary neuron cell treatment with 0.3 nM CuB with/without 1 ng/mL NGF for 72 hours significantly increased. The average of neurite length and primary dendrite number in each group is displayed in Figure 2B‐C. CuB at 0.3 nM markedly increased the neurite length of primary neuron cells from 40.1 ± 3.1 to 53.5 ± 3.8 µm and primary dendrite number from 2.7 ± 0.1 to 3.4 ± 0.2 (*P* < 0.01, *P* < 0.001). Moreover, 0.3 nM CuB‐combined 1 ng/mL NGF increased the neurogenesis effect to a level comparable with that of 10 ng/mL NGF (*P* < 0.001, *P* < 0.001). Collectively, these results suggest that CuB exerts a neurogenesis effect not only in PC12 cells but also in primary cortical neuron cells.

### 3.2 CuB increases newborn neurons of the hippocampus in ICR and APP/PS1 mice and rescues working memory in APP/PS1 mice

**Figure 3:**
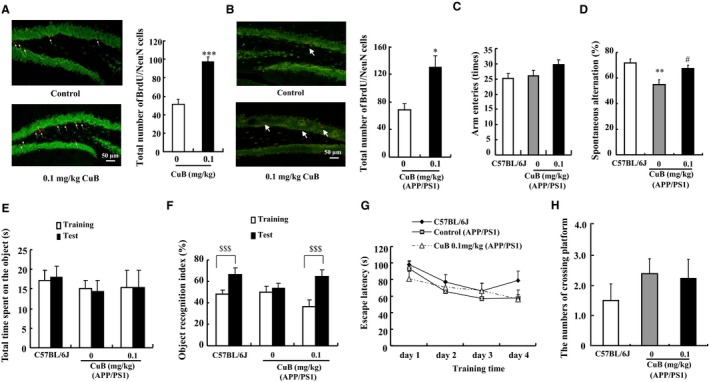
Neurogenesis effect of cucurbitacin B (CuB) on ICR and APP/PS1 mice. A‐B, Fluorescent immunohistochemistry image of 28‐day‐old newborn mature neurons stained with a BrdU antibody (red) and a NeuN antibody (green) of ICR and APP/PS1 mice, separately (n = 6). Scale bar is 50 µm. Bar graph showed the number of BrdU and NeuN double‐labelled cells. C‐D, The total arm entries and spontaneous alternation in Y‐maze test. E‐F, Time spent on object and object recognition index in NOR test (n = 8). G‐H, the escape latency and the number of crossing platform at day 5 in water maze experiment. The data represented mean ± SEM. *P < 0.05, **P < 0.01 and ***P < 0.001 indicated significant difference compared with the control group; ^#^ *P* < 0.05 indicated significant difference compared with the APP/PS1 control group; ^$$$^ *P* < 0.001 indicated significant difference between training and test sessions

The neurogenesis effect of CuB was further assessed in ICR normal mice in vivo. First, we did an experiment to confirm suitable concentration of CuB. Result in Figure S1B illustrated that the numbers of juvenile cells in the hippocampal DG zone of CuB 0.1 and 0.5 mg/kg groups were markedly increased compared with the control mice (*P* < 0.05, *P* < 0.05). Considering the security, we used 0.1 mg/kg CuB to perform the BrdU/NeuN double stain. The total number of 28‐day‐old BrdU/NeuN cells in 0.1 mg/kg CuB group was significantly higher than that in the control group (*P* < 0.001, Figure 3A). These results imply that CuB can increase juvenile cells and newly differentiated neuron cells in the hippocampus of normal mice.

The neurogenesis and neuroprotection effects of CuB were further studied on APP/PS1 mice, an AD model mouse with the method shown in Figure S1C. The total number of newly differentiated neuron cells in 0.1 mg/kg CuB group was significantly increased compared with APP/PS1 control group, as shown in Figure 3B (*P* < 0.05). Furthermore, animal behaviour tests were performed, and the results are presented in Figure 3C‐H. In the Y‐maze test, no significant differences in the number of arm entries were observed among these three groups (Figure 3C). The spontaneous alternation in the APP/PS1 group (58.9 ± 3.9%) was significantly decreased compared with the C57BL/6J group (71.5 ± 3.6%) (Figure 3D, *P* < 0.01). CuB at 0.1 mg/kg significantly improved spontaneous alternation in APP/PS1 mice to 67.2 ± 2.7% (Figure 3D, *P* < 0.05). Furthermore, NOR was performed to evaluate the memory. The total time spent on the object on training and test sessions of the three groups showed no significant differences (Figure 3E). However, in the test session, the object recognition index of C57BL/6J control group (66.5 ± 5.9%) and CuB + APP/PS1 group (65.0 ± 6.1%) was significantly higher than that of the APP/PS1 group (54.0 ± 4.4%) (Figure 3F). However, in the WMM, the treatment of CuB did not ameliorate the long‐term memory deficit of APP/PS1 mice (Figure 3G‐H). These results suggest that CuB can improve the working memory but not the long‐term memory of APP/PS1 mice.

### 3.3 CuB could modulate the GR/phospholipase C (PLC)/protein kinase C (PKC) and GR/ERK signalling pathway

**Figure 4:**
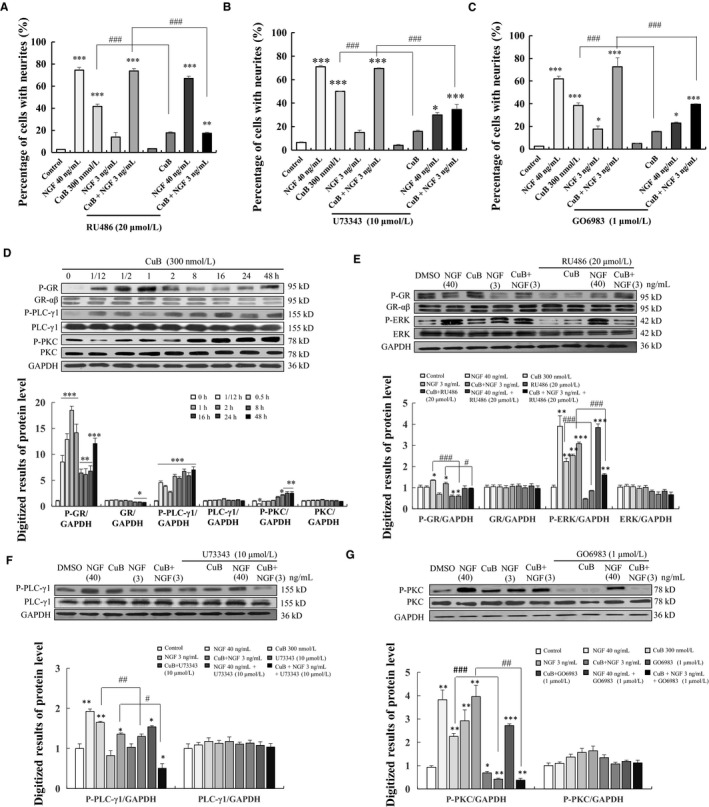
Effects of cucurbitacin B (CuB) on GR/PLC/PKC signalling pathway in PC12 cells. A‐C, Effects of GR inhibitor RU486, PLC inhibitor U73343 and PKC inhibitor GO6983 on CuB‐induced neurite outgrowth on PC12 cells. D, CuB stimulated the phosphorylation of GR, PLC‐γ1 and PKC in a time‐dependent manner. E, CuB stimulated the phosphorylation of GR and ERK (the cells were treated with each agent for 1 h), and GR inhibitor RU486 decreased the protein level of phosphorylation of GR and ERK in PC12 cells. F, PLCγ1 inhibitor U73343 attenuated the phosphorylation of PLCγ1 induced by CuB in PC12 cells (the cells treated with each agent for 16 h). G, PKC inhibitor GO6983 attenuated the phosphorylation of PKC induced by CuB in PC12 cells (the cells treated with each agent for 16 h). Each experiment was repeated for three times. The data represented mean ± SEM. *P < 0.05, **P < 0.01 and ***P < 0.001 indicated significant difference compared with the control group; ^#^ *P* < 0.05, ^##^ *P* < 0.01 and ^###^ *P* < 0.001 indicated significant difference compared with CuB or CuB with NGF group

Glucocorticoid receptor is involved in regulating neuronal structure and plasticity.^18^ Thus, the GR inhibitor RU486 was first used to illuminate the mechanism of CuB in PC12 cells. RU486 could significantly attenuate the percentage of PC12 cells with neurite outgrowth induced by 300 nM CuB from initial value of 41.5 ± 3.5% to 17.7 ± 0.9% and shorted the lengths (Figure 4A and Figure S3). Meanwhile, as PLC/PKC is located downstream of GR, the signalling is critical for the survival of sympathetic neurons.^19^, ^20^ The specific PLC inhibitor U73343 and PKC inhibitor GO6983 were used to examine the effects of CuB on the PLC/PKC signalling pathway. The neurite outgrowth of PC12 cells induced by treatment with CuB and CuB‐combined NGF was markedly reduced by U73343 and GO6983 (Figure 4B‐C, *P* < 0.001, *P* < 0.001, *P* < 0.001, *P* < 0.001). Subsequently, the phosphorylation of GR, PLC‐γ1 and PKC induced by 300 nM CuB at the protein level was investigated at multiple time points. The phosphorylation of GR protein was first increased, peaked at 1 hour and then decreased after 8 hours (Figure 4D). The phosphorylation levels of PKC and PLC induced by 300 nM CuB were initiated at 8 hours and then maintained for 48 hours (Figure 4D). Furthermore, the increase in GR and ERK phosphorylation in the 300 nM CuB with/without 3 ng/mL NGF groups could be significantly reduced by RU486 (Figure 4E and Figure S7B). A binding assay was carried out to determine whether CuB was potential GR ligand. However, the result showed that GR protein was not the target of CuB (Figure S4). Moreover, the phosphorylation of PLC‐γ1 and PKC in the 40 ng/mL NGF, 300 nM CuB and 300 nM CuB combined 3 ng/mL NGF groups was significantly decreased after treatment with U73343 and GO6983 (Figure 4F‐G and Figure S7C‐D). These results suggest that GR/PLC‐γ1/PKC signalling pathway is involved in the CuB‐induced neuronal differentiation of PC12 cells.

### 3.4 CuB could modulate the cofilin and actin signalling pathway

**Figure 5:**
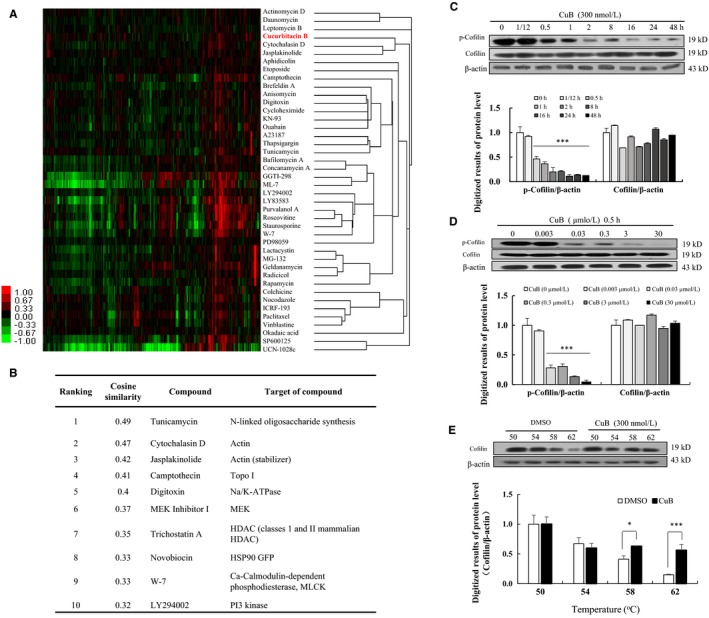
ChemProteoBase profiling result of cucurbitacin B (CuB) on HeLa cells and effect of CuB on the cofilin protein on PC12 cells. A, Hierarchical clustering of CuB and 41 well‐characterized compounds in ChemProteoBase was performed. B, Cosine similarity between CuB and compounds in ChemProteoBase was calculated, and top 10 compounds similar to CuB in ranking are shown. C, The protein‐level changes in p‐cofilin, cofilin and β‐actin after treatment with CuB at a dose of 300 nM for 48 h in PC12 cells. D, The protein‐level changes in p‐cofilin, cofilin and β‐actin after treatment with CuB at 0, 0.003, 0.03, 0.3, 3 and 30 µM for 30 min. E, The correlation of binding ability between CuB and cofilin protein at 50, 54, 58 and 62°C. The cells were treated with 300 nM CuB for 0.5 h. Each experiment was repeated for three times. The data represented mean ± SEM. *P < 0.001 and ***P < 0.001 indicated significant difference compared with the control group

Considering that the GR protein is not the target of CuB (Figure S4), we performed a hierarchical cluster analysis in HeLa cells to predict the probable target of CuB. The results are displayed in Figure 5A. The top 10 compounds in the ChemProteoBase are provided in Figure 5B. Compounds similar to CuB were not found in the data set, but compounds that ranked second or third were actin inhibitors, cytochalasin D (actin depolymerizer) and jasplakinolide (actin stabilizer). Some spots of actin significantly increased in the CuB‐treated HeLa cells. Similarly, previous studies reported that CuB could bind to cofilin, inducing cofilin hyperactivation and actin aggregation.^21^ Thus, the change in cofilin phosphorylation after adding CuB was investigated in PC12 cells. As expected, cofilin phosphorylation in CuB‐treated group was significantly decreased from 0.5 hour and lasted for 48 hours with increase in incubation time. Furthermore, the phosphorylation of cofilin was dose‐dependently decreased after adding CuB at doses of 0.003, 0.03, 0.3 and 3 to 30 μM (Figure 5C‐D and Figure S7E‐F). To confirm that if there was a binding correlation between CuB and cofilin protein, we performed CETSA using PC12 cell lines. As the temperature increase, the protein level of cofilin in CuB‐treated group was significantly higher than that of control group at 58 and 62°C (Figure 5E and Figure S7G, *P* < 0.05, *P* < 0.001). Therefore, these results further indicated that cofilin was targeting protein of CuB to produce NGF mimic and enhancer effects in PC12 cells.

### 3.5 CuB could modulate the TrkA/Ras/Raf/ERK signalling pathway

**Figure 6:**
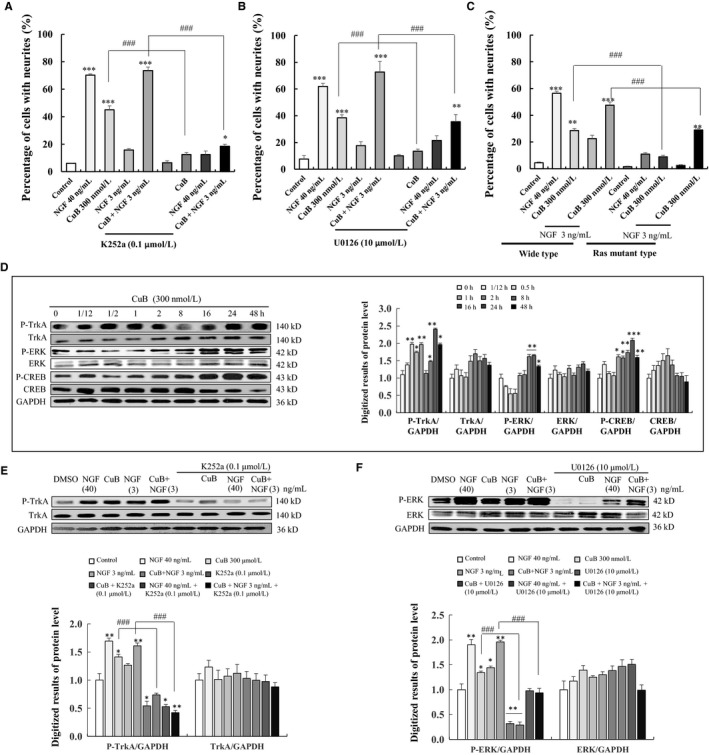
Effects of cucurbitacin B (CuB) on TrkA/ERK signalling pathway on PC12 cells. A‐B, Effects of TrkA inhibitor K252a, and ERK inhibitor U0126 on CuB‐induced neurite outgrowth on PC12 cells. C, The percentage of neurite outgrowth in wide‐type or Ras mutant‐type PC12 cells induced by CuB for 48 h. D, CuB stimulated the phosphorylation of TrkA, ERK and CREB in a time‐dependent manner. E, CuB stimulated the phosphorylation of TrkA (the cells were treated with each agent for 30 min) and inhibitor K252a decreased the protein level of phosphorylation of TrkA in PC12 cells. F, ERK inhibitor U0126 attenuated the phosphorylation of ERK induced by CuB in PC12 cells (the cells treated with each agent for 16 h). Each experiment was repeated for three times. The data represented mean ± SEM. *P < 0.05, **P < 0.01 and ***P < 0.001 indicated significant difference compared with the control group; ^#^ *P* < 0.05, ^##^ *P* < 0.01 and ^###^ *P* < 0.001 indicated significant difference compared with CuB or CuB with NGF group

TrkA could be stimulated by NGF in PC12 cells and lead to the formation of signalling endosomes.^22^ Thus, we examined whether the TrkA signalling pathway was taken part in the NGF mimic effect of CuB. The specific inhibitor of TrkA (K252a) could significantly attenuate the percentage of neurite outgrowth of PC12 cells induced by CuB from 45.0 ± 2.9% to 12.5 ± 1.5% and the neurite length of PC12 cells from 180.1 ± 14.6 to 100.1 ± 12.9 µm (Figure 6A and Figure S5, *P* < 0.001, *P* < 0.001). Similarly, K252a could decrease the neurite length and outgrowth of CuB‐combined NGF group (*P* < 0.001). Several signalling cascades initiated by TrkA receptor activation, including the Ras/Raf/ERK pathway, have been implicated in the NGF‐induced differentiation of PC12 cells.^22^ Thus, the effect of CuB on the Ras/Raf/ERK signalling pathway was investigated. As displayed in Figure 6B and Figure S6B‐C, the addition of Ras inhibitor (S3131), Raf inhibitor (AZ628) and ERK inhibitor (U0126) markedly reduced the percentage of neurite outgrowth of PC12 cells induced by CuB and CuB‐combined NGF at 48 hours, respectively. Furthermore, Ras mutant type of PC12 cells was used to detect the effect of CuB on Ras protein. CuB did not exhibit neurogenesis ability in Ras mutant PC12 cells (Figure 6C).

Then, the effects of CuB on TrkA/Ras/Raf/ERK phosphorylation were investigated at protein level. TrkA phosphorylation was significantly increased after treating CuB for 30 minutes (Figure 6D). The increase in ERK and CREB phosphorylation induced by CuB was begun at 8 hours and maintained for 48 hours (Figure 6D). Meanwhile, K252a at 0.1 μM could significantly decrease the TrkA phosphorylation in CuB‐treated groups and NGF‐treated group (Figure 6E and Figure S7I). However, the TrkB inhibitor ANA‐12 could not decrease the percentage of neurite outgrowth caused by CuB (Figure S6A). ERK phosphorylation in CuB and CuB‐combined low‐dose NGF groups was significantly decreased after treatment with U0126 for 16 hours (Figure 6F and Figure S7J). These results demonstrate that TrkA/Ras/Raf/ERK plays an important role on CuB‐induced neurite outgrowth on PC12 cells.

## 4 DISCUSSION

Cucurbitacin B has long been used as a liver protectant in China. A large number of studies have focused on its anti‐inflammatory, antioxidant, antiviral, antipyretic, analgesic and anti‐malaria activities.^8^ In the current study, we provided evidence in PC12 cells (Figure 1), primary neuron cells (Figure 2), ICR mice and APP/PS1 mice (Figure 3A‐B) that CuB also exhibits a neurogenesis function. Furthermore, animal behaviour tests showed that CuB could improve memory in APP/PS1 mice (Figure 3C‐F). Unlike the most popular acetylcholinesterase inhibitor anti‐AD drugs, CuB could ameliorate the memory deficits associated with enhanced neurogenesis.

Neuron loss is an important symptom of AD and is the main course of studying ability decline. Researchers found that increased newborn mature neuron might be a valuable method to improve learning and memory.^23^ In the present study, the memory protection effect of CuB on AD model mice was tested using Y‐maze, NOR and Morris water maze. Consistent with our inference, the results of Y‐maze and NOR tests showed that CuB could improve the working memory of AD mice (Figure 3C‐F). However, CuB did not ameliorate long‐term memory in the Morris water maze (Figure 3G‐H). At this point, the memory protective effect of CuB might only occur in the regulation of working memory but not long‐term memory.

The signalling pathways of CuB, including JAK2/STAT3, cofilin, cyclins and MAPK/ERK, have been reported.^8^, ^24^, ^25^ Glucocorticoids (GCs) can induce significant changes in the function of both neuronal and non‐neuronal cells. GCs can affect the structure and function of the CNS, especially those of the hippocampus.^26^, ^27^ In PC12 cells, GR is profoundly affected by neurite outgrowth and neurons.^18^ In the current study, CuB could modulate the GR/PLC/PKC and GR/ERK signalling pathways (Figure 4). However, the results of LanthaScreen™ TR‐FRET competitive binding assay showed that GR was not the direct target of CuB (Figure S4). Thus, hierarchical cluster analysis, a target prediction experiment, was conducted in HeLa cells to find the potential target of CuB (Figure 5). Results revealed that CuB could affect actin dynamics as an actin inhibitor in HeLa cells. However, several researchers found that the CuB‐induced actin aggregation by the formation of cofilin‐actin rods depended on cofilin hyperactivation.^28^ Cofilin, an actin‐depolymerizing factor, can sever actin filaments (F‐actin) when it is activated through dephosphorylation at the conserved serine residue 3.^29^ Thus, Western blot was performed to check the change in the upstream signalling pathway of actin in PC12 cells. Results in Figure 5C demonstrated that cofilin was hyperactivated by CuB in a time‐ and dose‐dependent manner, which agreed with the results of a previous study using A375 cells.^28^ We demonstrated that cofilin hyperactivation might be related to the events of CuB‐induced actin aggregation. Furthermore, we used the CESTA to investigate the correlation between cofilin and CuB. The result in Figure 5E indicated that cofilin might be the target protein of CuB to produce neurogenesis effect.

In the present study, we found that CuB could significantly increase the phosphorylation of PLC‐γ1 and dephosphorylation of cofilin to produce NGF mimic and enhancer effects in PC12 cells. Recently, some evidence also indicated that PLC‐γ1 signalling could regulate the localization of the actin‐depolymerizing and actin‐severing factor cofilin in the maintenance of dendritic spine morphology in brain,^30^ and its inhibitor U73122 could block the dephosphorylation and translocation of cofilin.^31^ This evidence supported our results which the interaction of PLCγ1 and cofilin took important roles during CuB produced NGF mimic and enhancer.

Some studies suggest that aside from the GR and cofilin signalling cascades, the TrkA/ERK pathway also affects differentiation.^32^ Therefore, we investigated the possible involvement of the TrkA/ERK pathway in regulating the NGF‐mimic effects of CuB. Results in Figure 6 suggested that TrkA also contributes to the NGF‐mimic effects of CuB. Meanwhile, Ras mutant PC12 cells could inhibit the differentiation of PC12 cells induced by CuB (Figure 6C). These results suggest that the NGF‐mimic effects of CuB on PC12 cells are mediated by TrkA receptor, and these effects activate ERK and CREB signalling cascades to induce neurogenic activities. TrkB was also investigated but was found to be not involved in CuB‐initiated cascades (Figure S6A). Notably, a major contribution of GR significantly induced ERK phosphorylation (Figure 4D), which was similar to a part of the TrkA signalling pathway. Thus, we inferred that several signalling pathways participate in the regulation of the NGF‐mimic effects of CuB.

In the current study, we confirmed that the neurogenesis effect of CuB was related to TrkA and GR. Interestingly, we found that several different type compounds could induce neurogenic activities through different signalling pathways such as tetradecyl 2,3‐dihydroxybenzoate and Lindersin B.^12^, ^32^ Lindersin B could induce neurite outgrowth via the TrkA signalling pathway. However, tetradecyl 2,3‐dihydroxybenzoate showed neuroprotective effect on PC12 cells by IGF‐1 receptor. Considering all of these results, we speculate that using a mixture of small molecules with different targets might strikingly increase the neurogenesis effect in PC12 cells, which is worth further studying.

**Figure 7:**
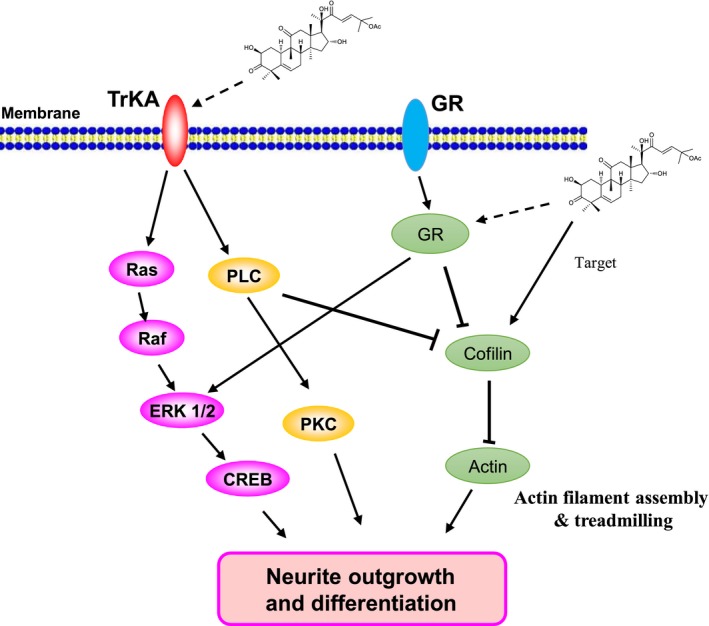
Proposed mechanism of cucurbitacin B (CuB) in induction of neurite outgrowth in PC12 cells. CuB might induce neurite outgrowth in PC12 cells via activation of TrkA/ERK and GR/PLC/PKC signalling pathways, and cofilin might be the candidate target protein of CuB in PC12 cells

In summary, CuB can enhance neurogenesis in vitro and in vivo and ameliorate the working memory of APP/PS1 mice. In PC12 cells, cofilin might be the candidate target of CuB, and the GR/PLC/PKC and TrkA/Ras/Raf/ERK signalling pathways play important roles in the neuroprotection of CuB (Figure 7). As CuB is already in clinical use, it could be a novel therapeutic for Alzheimerr's disease. Further work is warranted to reveal the direct target(s) of CuB, which may ultimately explain its mechanism.

## CONFLICT OF INTEREST

The authors confirm that there are no conflicts of interest.

## AUTHOR CONTRIBUTIONS

J. Qi and L. Xiang supervised the research; J. Li, K. Sun and L. Gao performed the experiments; H. Osada and M. Muroi designed and conducted the ChemProteoBase profiling study; H. Osada and Y.‐T. Chang provided materials; J. Li, L. Xiang, H. Osada and J. Qi wrote the manuscript.

## Data Availability

The data that support the findings of this study are available from the corresponding author upon reasonable request.
