# Supplementary material for: Cucurbitacin B induces neurogenesis in PC12 cells and protects memory in APP/PS1 mice
Source: J Cell Mol Med. 2019 Jun 30;23(9):6283–94. doi: 10.1111/jcmm.14514 (PMC6714235; doi:10.1111/jcmm.14514)
Supplement: Supplementary file 1 [file JCMM-23-6283-s001.docx]

**Supporting Information**

**Cucurbitacin B induces neurogenesis in PC12 cells and protects memory in APP/PS1 mice**

Jing Li^1^, Kaiyue Sun^1^, Makoto Muroi^2^, Lijuan Gao^1^, Young-Tae Chang^3-4^, Hiroyuki Osada^2^, Lan Xiang^1^*, Jianhua Qi^1^*

^1^College of Pharmaceutical Sciences, Zhejiang University, Hangzhou 310058, China

^2^Chemical Biology Research Group, RIKEN Center for Sustainable Resource Science, 2-1 Hirosawa, Wako, Saitama 351-0198, Japan

^3^Center for Self-assembly and Complexity, Institute for Basic Science (IBS), Pohang 37673, Republic of Korea

^4^Department of Chemistry, Pohang University of Science and Technology (POSTECH), Pohang 37673, Republic of Korea

***Corresponding author:**

Jianhua Qi email: qijianhua@zju.edu.cn, Tel/Fax: +86-571-88208627

Lan Xiang email: lxiang@zju.edu.cn, Tel/Fax: +86-571-88208627

**Materials and Methods**

Isolation and structure elucidation of CuB

The stems of *C. melo* were purchased from HuQingYuTang Pharmacy in Hangzhou, Zhejiang Province, China. Exactly 100 g of *C. melo* were first powdered and then extracted with 2.5 L of ethanol for 3 days at room temperature. The supernatant was separated by filtration and concentrated to obtain 23.3 g of crude ethanol extract. This extract of was partitioned between 80% aq. MeOH and *n*-hexane. The sample from the layer of 80% aq. MeOH was concentrated (4.6 g), chromatographed on a silica gel open column (200–300 mesh, Yantai Chemical Industry, Shandong, China), and eluted with *n*-hexane/EtOAc gradient at ratios of 90:10, 80:20, 70:30, 40:60, and 0:100 to yield 30 fractions. The fractions eluted with *n*-hexane/EtoAc (70:30) were collected (318.6 mg) and further purified by reverse-phase HPLC [Develosil ODS-UG-5 (20×250 mm), Nomura Chemical, flow rate: 2 mL/min, wavelength: 228 nm] with 65% aq. MeOH to produce a pure compound (retention time = 20.8 min, 217.0 mg). The chemical structure of the compound was determined to be CuB (Fig. 1A) by comparing ^1^H NMR and ^13^C NMR with the reported literature [1].

Cucurbitacin B: white solid, [α]22 D+57.2 (*c* 0.5, EtOH). ^1^H NMR (500 MHz, acetone-d_6_) 0.91, 1.02, 1.11, 1.28, 1.32, 1.39, 1.40, 1.44, 1.51, 1.55, 1.84, 1.96, 1.97, 2.11, 2.40, 2.51, 2.66, 3.02, 3.40, 3.68, 3.85, 4.45, 4.56, 5.82, 6.81, 6.98. And ^13^C NMR (125 MHz, acetone-d_6_): 19.22, 20.24, 20.61, 21.81, 21.96, 24.66, 25.12, 26.42, 27.06, 29.92, 34.16, 37.10, 43.55, 46.56, 48.98, 49.14, 49.44, 51.11, 51.41, 59.07, 71.35, 72.31, 79.57, 80.12, 120.79, 122.25, 141.97, 151.01, 170.27, 203.47, 212.72, 213.72. ESI-MS m/z 581.3085, [M+Na]^+^.

MTT Assay

In MTT assay, cells were first cultured in a 24-well microplate in 1 mL serum-free DMEM medium containing 0.5% DMSO or a test sample for 48 h. The medium was carefully removed by aspiration, replaced with 0.5 mL fresh medium containing 0.2 mg/mL MTT, and then incubated at 37 °C for 2 h. To solubilize the formazan crystals, the medium was completely removed, and then 0.2 mL of DMSO was added to the well. The OD value of each well was detected at 570 nm using a microplate reader (Bio-Tek Instruments, VT, USA). Cell viability = (OD of experiment group - OD of blank)/(OD of DMSO group - OD of blank) × 100%. The experiments were repeated thrice.

Primary cortical neuron cells culture and bioassay

Primary cortical neuron cells were prepared from the embryos of ICR mice (Zhejiang Academy of Medical Sciences, Hangzhou, China) at embryonic day 18. The cortex was dissociated by 0.25% trypsin (Gibco, NY, USA) with 1% DNase (Sangon Biotech, Shanghai, China) at 37 °C for 20 min and stopped by DMEM containing 10% horse serum. Then 60,000 cortical cells per well were plated onto 24-well plates coated with 0.1 g/L poly-L-lysine (Sigma, MO, USA). Cells were grown in 1 mL neurobasal medium [96% neurobasal medium, 2% B-27 supplement, 1% GlutaMaxTM supplement (Gibco, NY, USA), 1% 100 U/mL penicillin-streptomycin (Beyotime Biotechnology, Shanghai, China)] at 5% CO_2_ and 37 °C. After 2 h, the 500 µL culture medium in each well was replaced with fresh neurobasal medium. After 24 h, 0.5% DMSO or test samples were added into the medium and the cells were continually incubated for three days. At the 3rd day after adding the samples, the cells were washed with PBS and incubated with 500 nM NeuO for 1 h as described in the report [2]. The micrograph change of the cells was determined under a phase-contrast fluorescence microscope (Leica DMI 3000B, Wetzlar, Germany) at an excitation of 488 nm in three arbitrary areas. The length of neurites and the number of extremities were analyzed by Image J (National Institutes of Health, MD, USA).

**Morris Water Maze (MWM) test**

The MWM was performed after the NOR test and the apparatus comprised a circular tank (1.2 m diameter, 50 cm depth), a 14 cm movable platform (submerged 1 cm below the water), a video camera, and a computer. Before the experiment, water was added and warmed it to 22 ± 1°C. The MWM test consisted of four-day training sessions and one-day testing session and the tank was divided into four quadrants. In the training sessions, the mice were placed facing the wall in the pool and learned to run away from the water by reaching the submerged platform within 120 s. If the mice could not locate the platform, they were guided and allowed to stay in the platform for 10 s. Each mouse was performed four trails for four quadrants in one day. The testing session were done on the fifth day without the platform, each mouse was placed in a same starting position and allowed to swim for 90 s. The time for each mouse to reach the target (escape latency) during the training sessions and the numbers of crossing the platform was recorded by a video camera and software.

Western blot

Briefly, 2×10^6^ PC12 cells were seeded and cultured in a 6 cm plate containing 5 mL of DMEM medium for 24 h. In the time-dependent study of CuB, 300 nM CuB was added and incubated for designed time. In the inhibitor study, the PC12 cells were first incubated with 4 mL of serum-free DMEM medium containing an inhibitor for 30 min and then added with 1 mL medium with test samples for designated hours. To prepare protein lysates, the cells were collected and lysed in lysis buffer. After centrifugation at 12,000 rpm for 15 min, the supernatant was removed. Protein concentration was determined by protein assay kit (ComWin Biotechnology, Beijing, China). Proteins (10 or 20 μg) of each group was separated by 10% SDS-PAGE gel and electrophoresed at 130 V for 60 min followed by transferred to the polyvinylidenedifluoride membranes at 350 mA for 75 min. The membrane was blocked with 5% nonfat milk for 1.5 h. Then the membrane was incubated with different first antibodies for 1 h in 2% non-fat milk followed by incubated with secondary antibody for 45 min after washing thrice with TBS. The bands were visualized using cECL Western blot kit (ComWin Biotechnology, Beijing, China), and density analysis was performed using Image J software. The first antibodies were obtained from the following sources: anti-p-TrkA, -TrkA, -p-ERK, -ERK, -p-PKC, -PKC, -p-PLC-γ, -PLC-γ, -p-CREB, -CREB, -p-cofilin, -cofilin (Cell Signaling Technology, MA, USA), anti-GAPDH, -β-actin (ComWin Biotechnology, Beijing, China), anti-p-GR (Affinity BioReagents, OH, USA), and anti-GR (Santa Cruz, CA, USA). The secondary antibodies horseradish peroxidase-conjugated anti-rabbit and anti-mouse IgGs were purchased from ComWin (Beijing, China).

LanthaScreen TR-FRET competitive binding assay

The LanthaScreen time-resolved fluorescence resonance energy transfer (TR-FRET) GR [competitive binding assay](https://www.sciencedirect.com/topics/medicine-and-dentistry/competitive-binding-assay) was used to determine if CuB was potential GR ligand and was carried out by the Thermofisher scientific company (Waltham, MA, USA). Briefly, A 10-point [serial dilution](https://www.sciencedirect.com/topics/medicine-and-dentistry/serial-dilution) of CuB and dexamethasone (control ligand) were prepared in [DMSO](https://www.sciencedirect.com/topics/medicine-and-dentistry/dimethyl-sulfoxide) before the experiment. Then, 4 μL of each test sample was added into a black 384-well assay plate followed by 8 μL mixture of 2 × GR-LBD(GST) and terbium-labeled anti-GST antibody dissolved in an assay buffer (TR-FRET coregulator buffer K, GR stabilizing peptide, and 5 mM DTT). Afterwards, 4 µL of 4 × fluormone GS1 Green tracer was added, and the plate was incubated at room temperature for 60 min. TR-FRET was measured by using the EnVision Multilabel Plate Reader (PerkinElmer, GA, USA). Emission Ratio (ER) was calculated by dividing the emission signal at 520 nm by 495 nm. The percentage of displacement was defined by (ER _0%_ _displacement_ - ER _Sample_)/(ER _0% displacement_ - ER _100%_ _displacement_) x 100. A binding curve was generated by plotting the displacement ratio vs concentration (nM) using GraphPad Prism software.

**Reference**

[1] **Wu PL, Lin FW, Wu TS, *et al.*** Cytotoxic and anti-HIV principles from the rhizomes of *Begonia nantoensis*. *Chem Pharm Bull.* 2004; 52: 345-349.

[2] **Er JC, Leong C, Teoh CL, *et al.*** NeuO: a fluorescent chemical probe for live neuron labeling, *Angew Chem.* 2015; 127: 2442-2446.

**Figures**

**
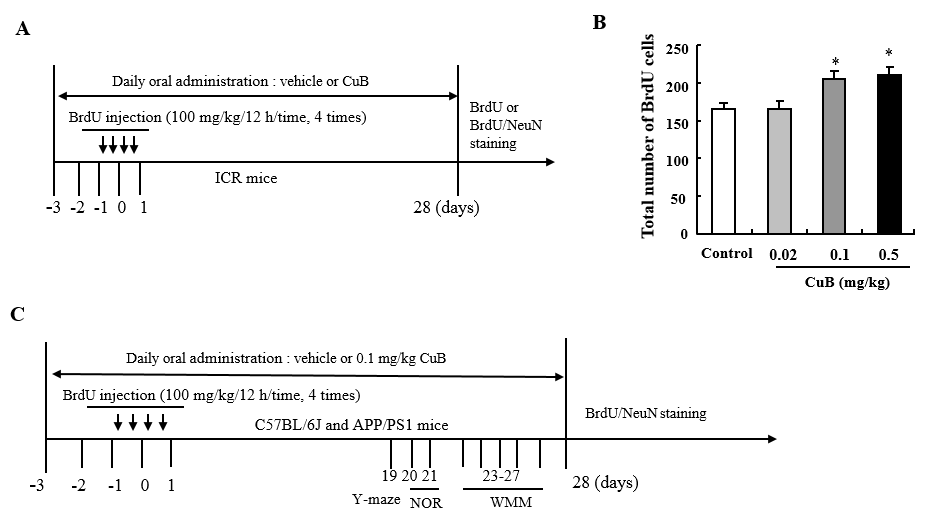
**

**Supplementary Figure 1.** A, The procedure of BrdU or BrdU and NeuN double immunohistochemistry in ICR mice (n = 6). B, The juvenile cells in bilateral hippocampal DG zone of ICR mice were stained with BrdU, and bar graph showed the total number of BrdU labeled cells in four groups. The data represented mean ± SEM (n = 6). *p<0.05 indicated significant difference compared with the control group. (**C)** The procedure of neuroprotection experiment of CuB on the APP/PS1 mice (n = 8).

**
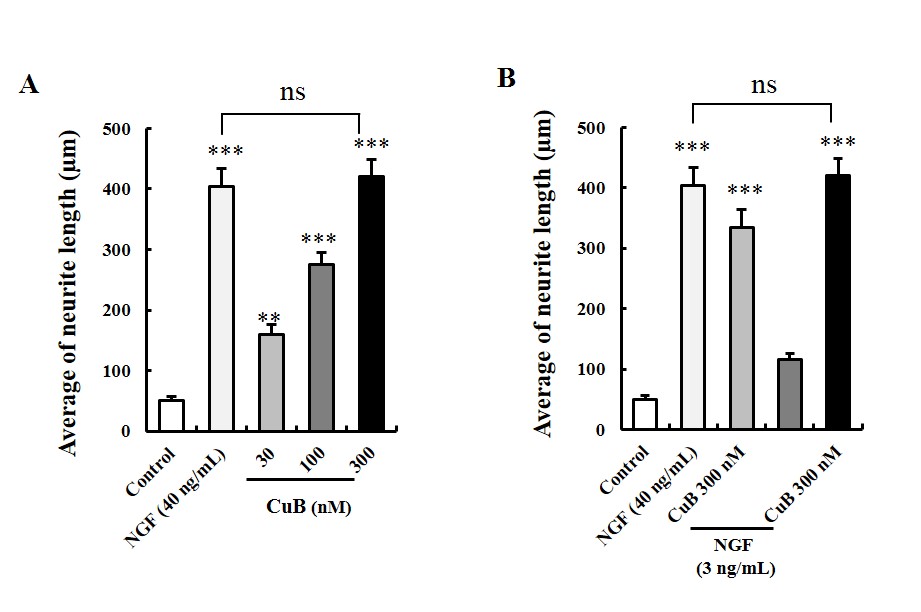
**

**Supplementary Figure 2.** A-B, Average of neurite length of PC12 cells after treated with CuB or NGF for 48 h. The experiment was repeated for three times. The data represented mean ± SEM. **p<0.01, ***p<0.001 indicated significant difference compared with the DMSO control group; ns indicated no significant difference compared with 40 ng/mL NGF group.

**
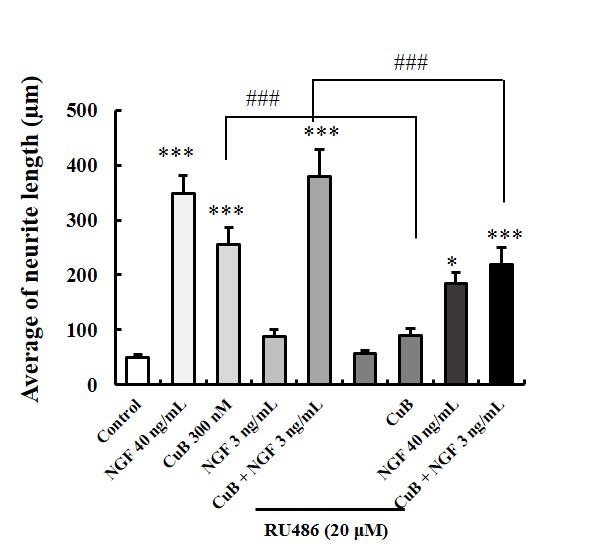
**

**Supplementary Figure 3.** Average of neurite length in each group in RU486 inhibitor test in PC12 cells. The experiment was repeated for three times. The data represented mean ± SEM. *p<0.05, ***p<0.001 indicated significant difference compared with the DMSO control group; ^###^p<0.001 indicated significant difference compared with 300 nM CuB or CuB + 3 ng/mL NGF group.

**
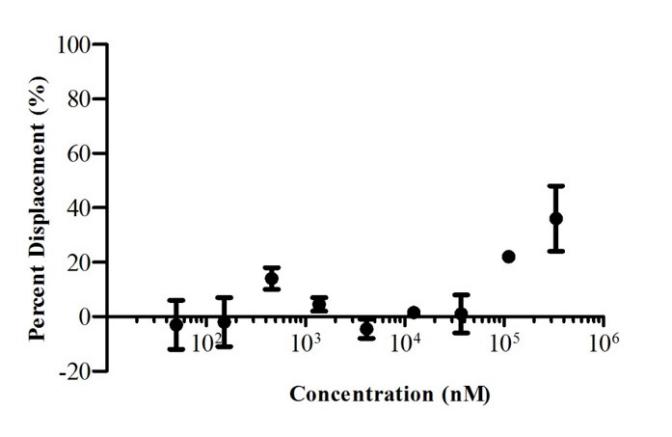
**

**Supplementary Figure 4.** The change of percent displacement (%) in GR [competitive binding assay](https://www.sciencedirect.com/topics/medicine-and-dentistry/competitive-binding-assay). The experiment was repeated for three times. The data represented mean ± SEM.

**
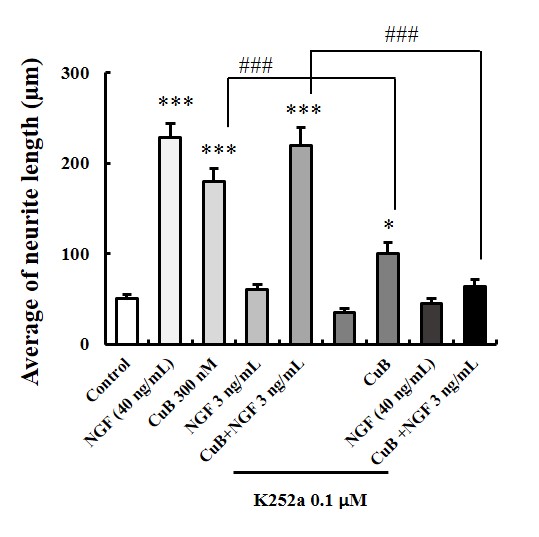
**

**Supplementary Figure 5.** Average of neurite length in each group in K252a inhibitor test in PC12 cells. The experiment was repeated for three times. The data represented mean ± SEM. *p<0.05, ***p<0.001 indicated significant difference compared with the DMSO control group; ^###^p<0.001 indicated significant difference compared with 300 nM CuB or CuB + 3 ng/mL NGF group.

**
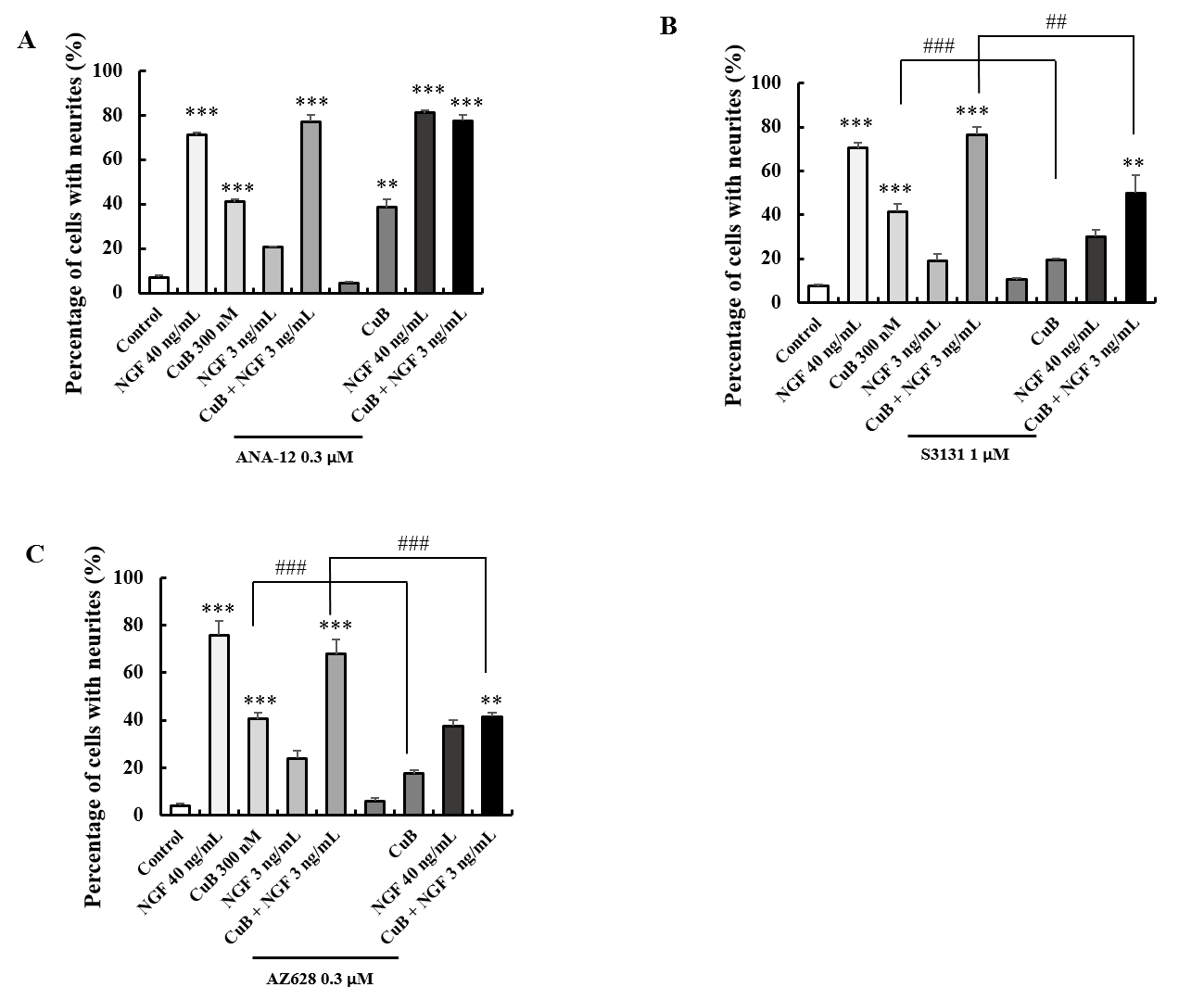
**

**Supplementary Figure 6.** A-C, Percentage of PC12 cells with neurites in each group in the ANA-12, S3131 and AZ628 inhibitor tests. Each experiment was repeated for three times. The data represented mean ± SEM. **p<0.01, ***p<0.001 indicated significant difference compared with the DMSO control group; ^##^p<0.001, ^###^p<0.001 indicated significant difference compared with 300 nM CuB or CuB + 3 ng/mL NGF group.


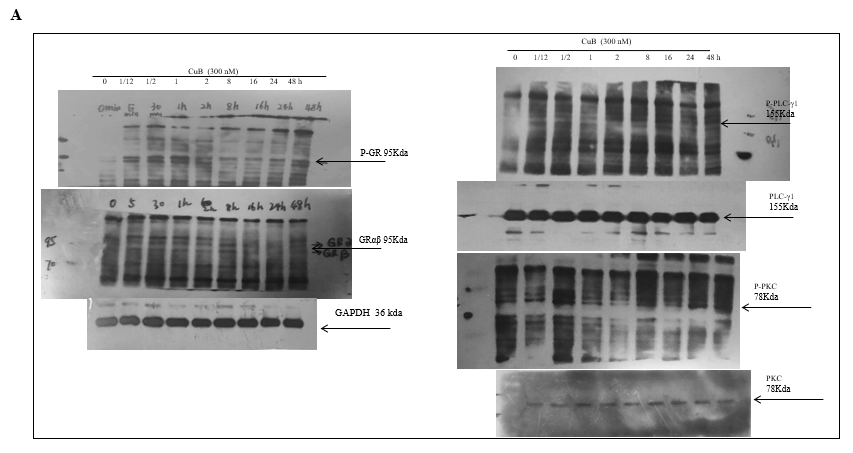


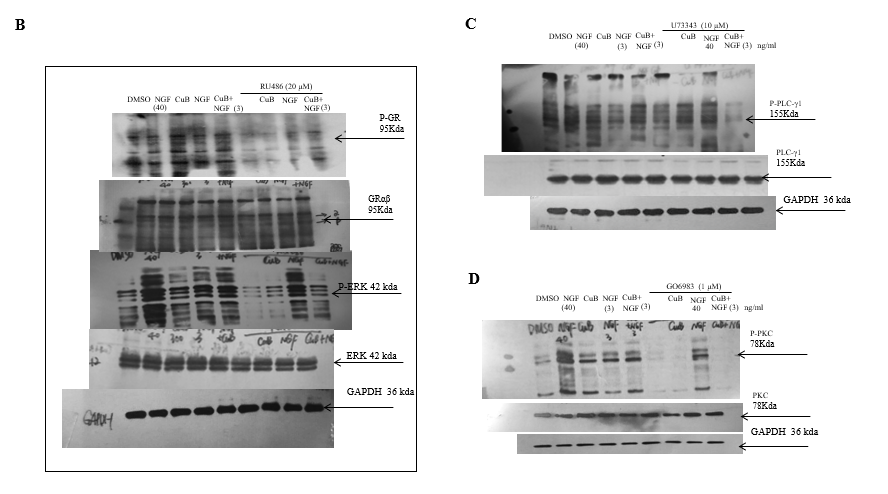


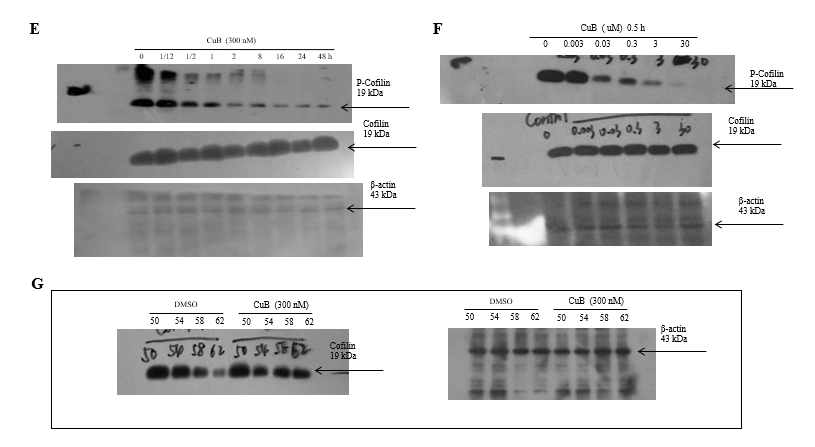


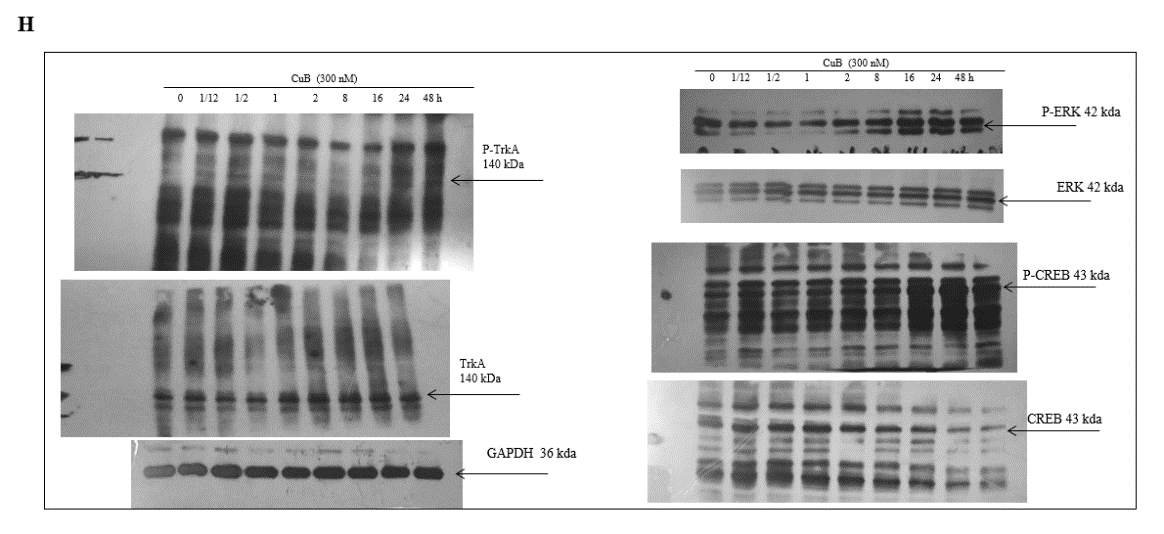


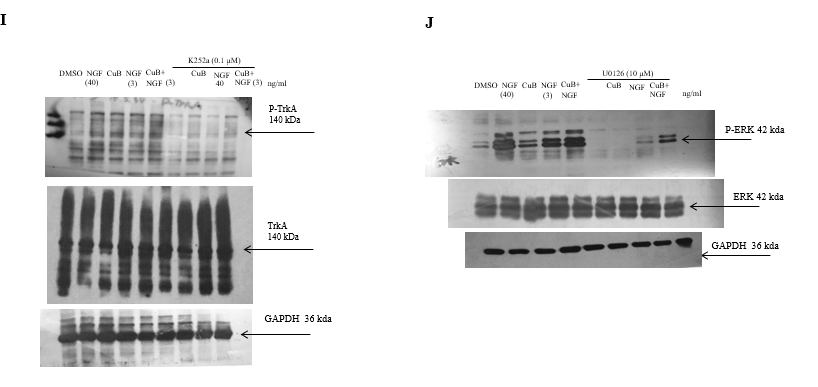


**Supplementary Figure 7.** Figures A-J presented the original figures of Western blots in figures 4D, 4E, 4F, 4G, 5C, 5D, 5E, 6D, 6E, 6F respectively.
